# Comparative Evaluation of Two Different Types of Abutments in Relation to Post-prosthetic Bone Resorption and Periodontal Pocket Depth: A Randomized Controlled Clinical Trial With a One-Year Follow-Up

**DOI:** 10.7759/cureus.25807

**Published:** 2022-06-10

**Authors:** Mohammad Anas Almodalal, Mhd. Luai Morad, Mohammad Y Hajeer, Munir Harfouche

**Affiliations:** 1 Department of Fixed Prosthodontics, University of Damascus Faculty of Dentistry, Damascus, SYR; 2 Department of Orthodontics, University of Damascus Faculty of Dentistry, Damascus, SYR; 3 Department of Oral and Maxillofacial Surgery, University of Damascus Faculty of Dentistry, Damascus, SYR

**Keywords:** depth of the periodontal pocket, bone loss, 3d laser printer, lost wax technique, molded abutment, printed abutment, standard abutment, dental implant

## Abstract

Background and aim: In the past few years, the number of studies conducted on the designs of abutments and related materials has increased. The restoration is performed over the implant using prefabricated standard abutments. Due to the problems encountered in relation to bone resorption following restoration, other types of abutment designs have been introduced in the dental field such as molded abutments usually fabricated by the lost-wax technique and computer-designed abutments produced by three-dimensional (3D) printing technology. The aim of this study was to compare the clinical performance of molded abutments and computer-designed 3D printed abutments in terms of the bone loss around the implant and the depth of periodontal pockets in a one-year observation period.

Methods: The research sample consisted of 32 dental implants for patients who required two adjacent implants. Patients were randomly allocated into two groups: in the first group, the patients received molded abutments, whereas, in the second group, the patients received 3D printed abutments with an allocation ratio of 1:1. In the molded abutment group, the plastic abutments were waxed and poured using the Ni-Cr mixture in the lost-wax technique, while in the printed abutment group, the abutments were designed by a computer program and printed using the Cr-Co mixture employing a 3D laser printer. The bone level and pocket depth around the implant were evaluated at 3, 6, and 12 months following cementation.

Results: After one year, there were significant differences in the bone resorption mean values between the 3D laser-printed abutment group (0.43±0.11) and molded abutment group (0.54±0.11). In addition, there were significant differences in the mean values of probing depth between the 3D laser-printed abutment group (3.39±0.12) and molded abutment group (3.53±0.08). Therefore, the 3D laser-printed abutment was slightly better and had a lower bone loss degree than the molded abutment.

Conclusion: Within the limitations of the current work, the mean bone resorption for both types of abutments was within the normal limits. However, the implants that were restored using the printed abutments had less bone resorption than those restored using the molded abutments.

## Introduction

Dental implants are an established treatment modality in dental practice and are used to restore the functional and aesthetic aspects of the edentulous patient [1]. To achieve the optimal results of dental implants at the functional and aesthetic levels, the implants must be applied at an ideal position in terms of length, diameter, and inclination, which increases the implant’s tolerance to occlusal forces and secures the cosmetic aspect as much as possible [2]. Recently, there has been an increase in the number of studies conducted on the design of dental implants and their abutments, the employed materials, and the techniques used to place and replace implants. Implant design and the implant-abutment interface have been regarded as key influences on bone maintenance over time [3].

The platform-switching concept is the use of an abutment smaller in diameter than the implant platform; the internal gap between the neck of the implant and the neck of the abutment is among other factors that determine implant performance and bone loss after restoration, including the dimensions of the loss area and the form of contact between the implant and the abutment [4-6].

Microbial leakage at the implant-abutment interface has been associated with inflammatory reactions that may jeopardize peri-implant bone stability [7]. The restoration over the implant is usually done using standard abutments pre-manufactured by the implant company, but due to the occurrence of bone absorption and an increase in the depth of the pocket after restoration, there is a risk of failure of the entire implant system [8]. The molded abutment was presented as an alternative solution to the standard abutment due to its low cost and the possibility of making the necessary adjustments to suit the cosmetic and functional needs, thereby eliminating the problems caused by the standard one. However, the problem with this abutment is the deformation caused by the casting process, which would affect the stability of the final restoration and the attachment of the abutment to the implant. This lack of precision may put the patient at a higher risk of implant failure due to peri-implant inflammation in the soft tissues around the implants and absorption into the surrounding bone [9,10].

Recently, three-dimensional (3D) printing technology has become more important in the world of dentistry, especially selective laser-melting metal printing technology [11,12]. With the rapid development and integration of software and hardware technologies, 3D printing technology has been used to fabricate high-quality restorations with greater accuracy, in addition to facilitating abutment design by clinicians, without intermediate production steps, which saves time and money [12,13]. The computer-designed abutment made by the 3D printer is specially designed for patients following their cosmetic and functional needs.

Most of the published articles that evaluated 3D printed abutments were only confined to laboratory investigations or short-term clinical studies [14]. It is recommended to conduct more clinical studies with a longer follow-up period of not less than one year to evaluate the clinical performance of these prostheses [15]. The aim of this research was to compare the amount of bone loss and depth of periodontal pockets between the traditional molded abutments and the computer-designed 3D printed abutments after 3, 6, and 12 months.

## Materials and methods

Study design and settings

This study was a parallel-group randomized controlled trial conducted at the Department of Fixed Prosthodontics, Faculty of Dentistry, Damascus University, Damascus, Syria. It was ethically approved by the Local Research Ethics Committee of the Faculty of Dentistry, Damascus University (UDDS-5455-02092020/SRC-5791). The project was self-funded and it was registered in the Clinical Trials database with the following registration number: NCT05350293.

Sample size calculation

G*Power software, version 3.1.3 (Heinrich-Heine-University, Düsseldorf, Germany) was used to calculate the sample size. Crestal bone loss was the variable of interest in this study. The following assumptions were used: (1) the smallest difference requiring detection of the crestal bone loss after one year was 1.5 mm; (2) a significance level of 0.05, (3) a power of 95%, (4) the standard deviation (SD) of the change in the crestal bone level was 0.33 mm, as taken from a previous study [16], (5) the intended inferential statistical approach was two-sample t-tests. The calculation revealed that a sample size of 16 patients was required for each group.

Patient recruitment and follow-up

Participants were derived from patients attending the Department of Fixed Prosthodontics of the University of Damascus Dental School, who required two adjacent or opposite implants (single posterior prosthesis) on each side of the jaw, whether upper or lower, and wished to have a prosthetic solution with implants. After the clinical, dental, and radiographic examination, 32 patients accurately met the following inclusion criteria as specified by the American Society of Anesthesiologists [17]. The inclusion criteria included the following: (1) age greater than 20 years and less than 50, (2) good oral health, and (3) absence of systemic diseases that affect the healing of surrounding tissues, such as diabetes.

Exclusion criteria were as follows: (1) the presence of non-functional habits such as stridor, (2) acute periodontitis, (3) previous loss of implants, (4) poor general health conditions, (5) previous radiotherapy in the head and neck area, (6) mental incompetence, and (7) orthodontic treatment.

Randomization

Randomization was performed using a computer-generated random list (Excel 2007, Microsoft Windows; Microsoft, Redmond, WA) with an allocation ratio of 1:1. The patients were randomly allocated into the two groups. In the first group, the patients received molded abutments, whereas, in the second group, the patients received 3D printed abutments. Using sealed and sequentially numbered envelopes, the allocation procedure was concealed from the researcher and was conducted by one of the academic staff not involved in this research project. Blindness was only applied during data analysis.

Implantation procedures

**Figure 1:**
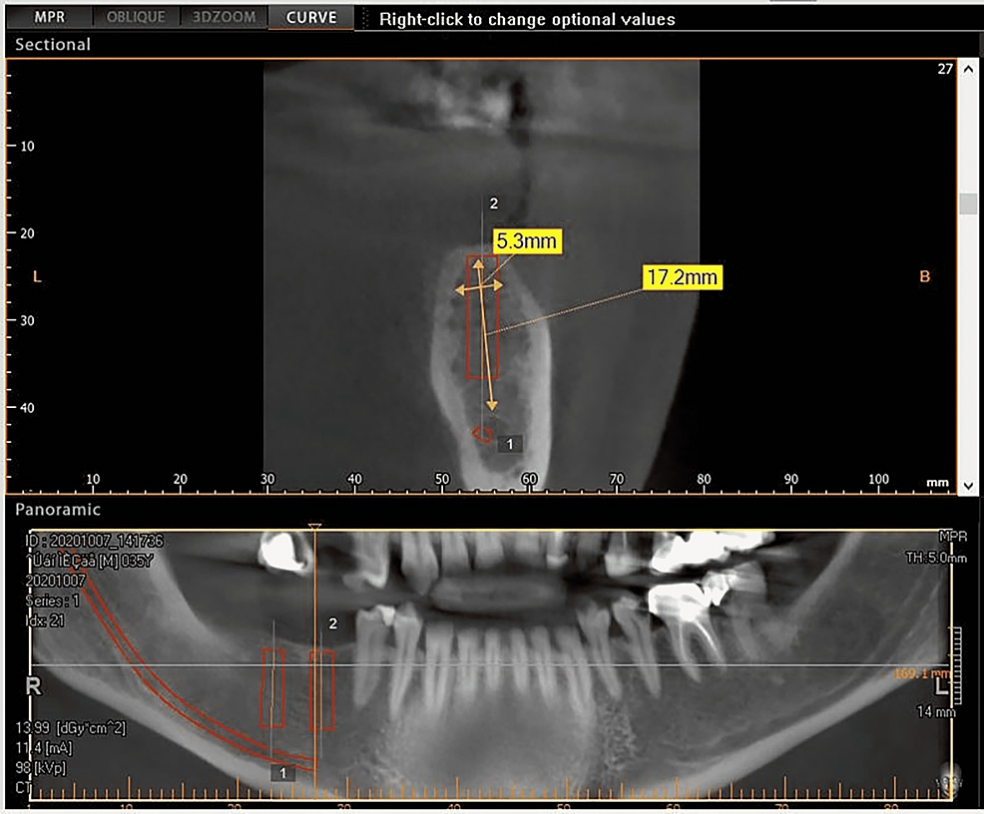
Planning implant position and determining the diameter and length of the implant using cone-beam computed tomography imaging

**Figure 2:**
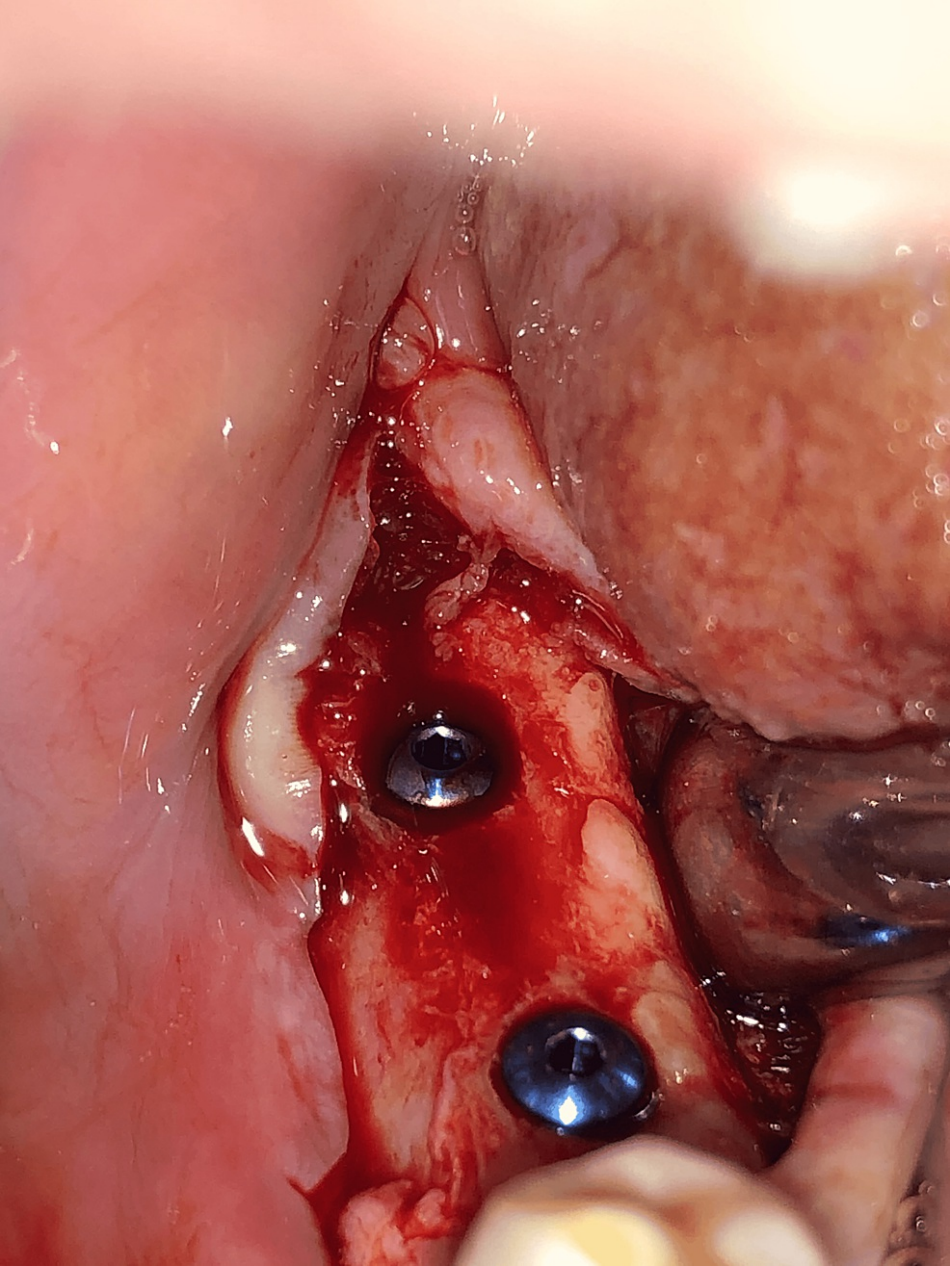
Placement of the implants in the alveolar bone with a 3-mm inter-implant distance

**Figure 3:**
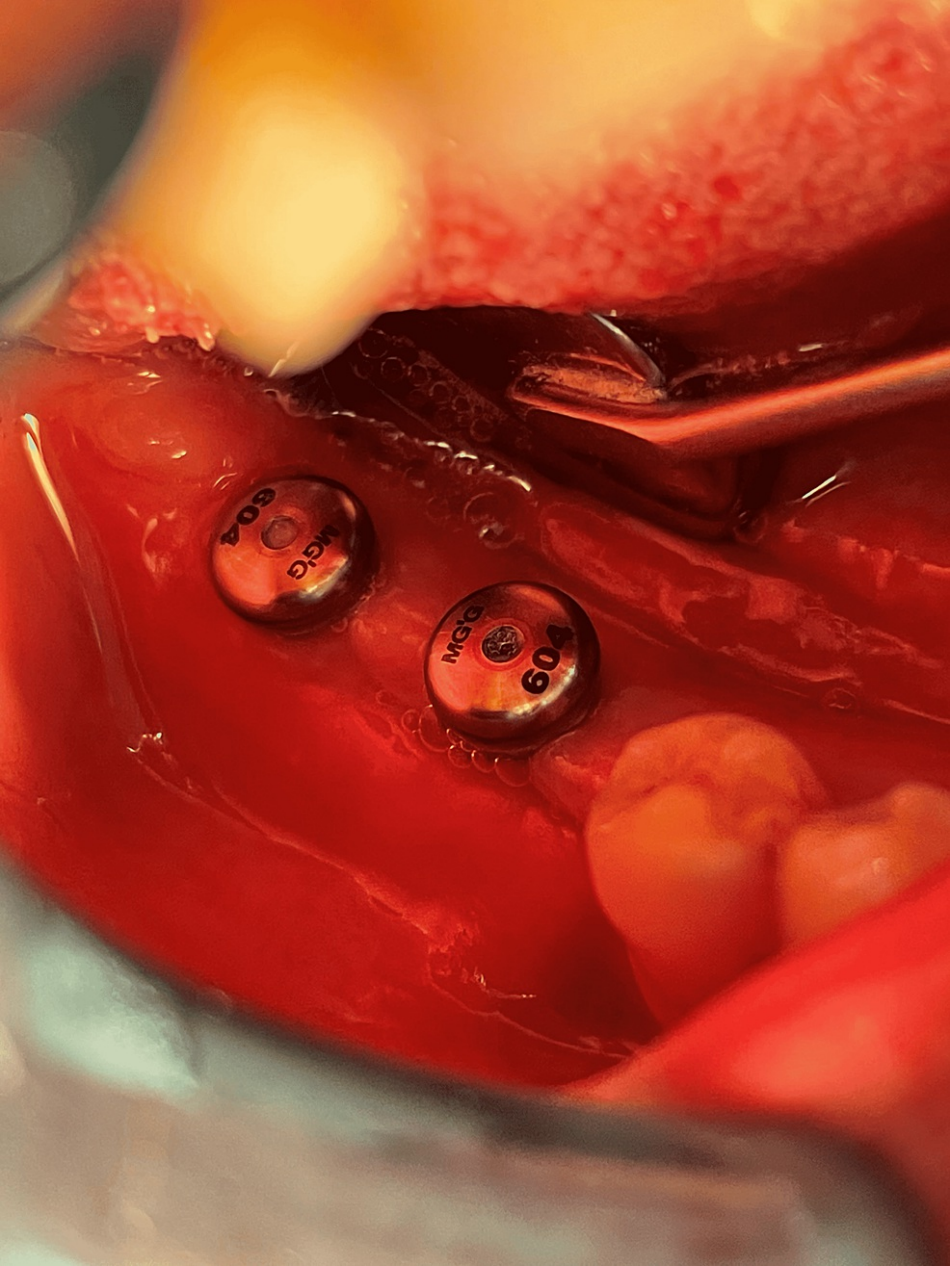
Placement of the gingival healing abutments

**Figure 4:**
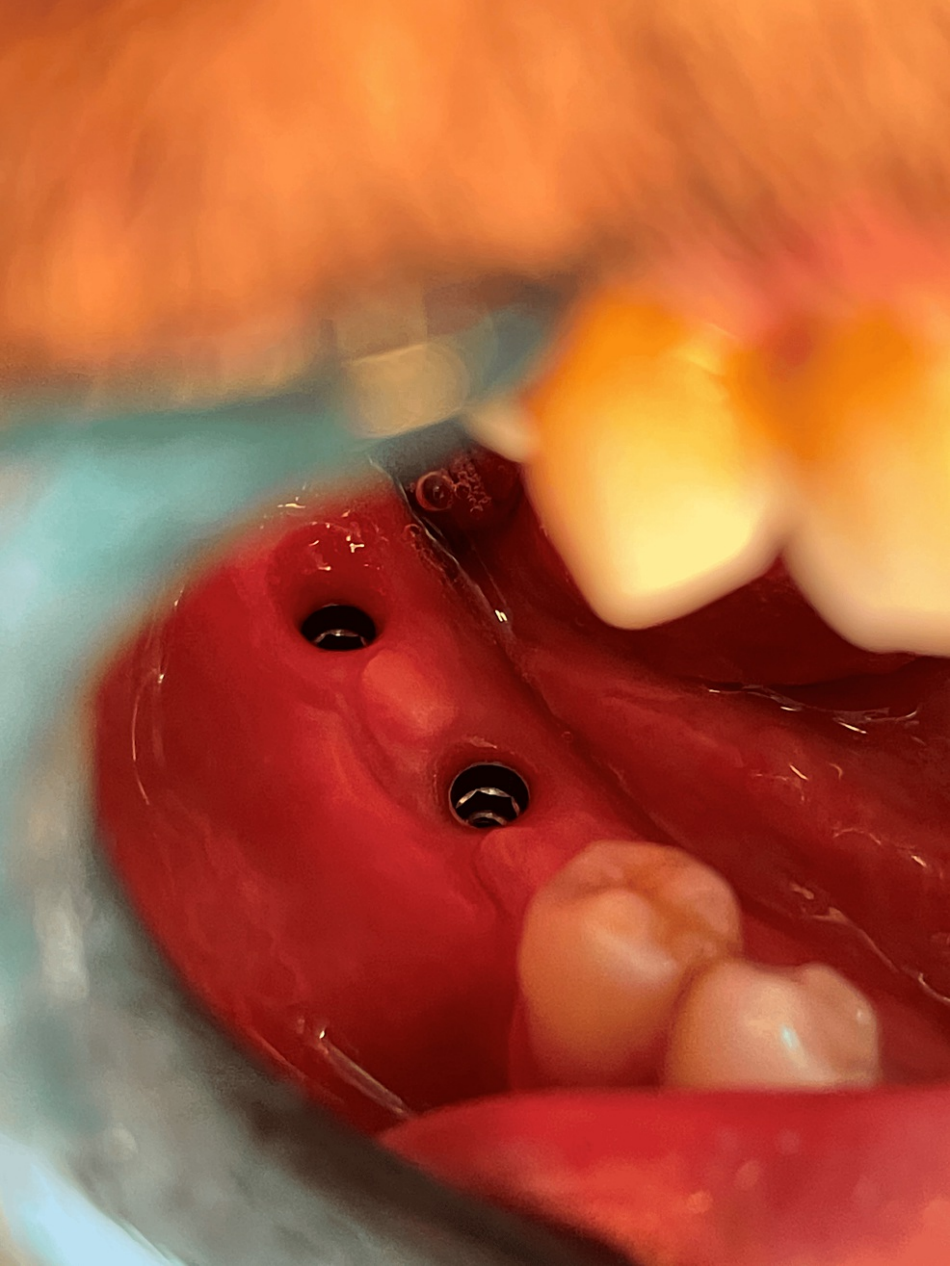
The emergence profile of the gingiva after using the healing abutments

The first surgical stage included inserting the implants (Any Ridge®; MegaGen, Seoul, South Korea) in the place of loss after conducting a radiological study using cone-beam computed tomography (CBCT) imaging that allowed knowing the type of bone and determining the diameter and length of the implant that was appropriate to the place of loss (Figures 1, 2). The second surgical stage included the detection of implant position and placement of the gingival healing abutment (Figures 3, 4).

Prosthetic stage

The impression was taken for the implants in the open-tray technique using the appropriate transfer and an alternative to the laboratory implant. The gingival mask was injected similarly to placing the gum inside the mouth. The impression was poured using phosphate-bonded gypsum powder (Maruvest Speed; Megadental, Büdingen, Germany).

First group: plastic molded abutments

**Figure 5:**
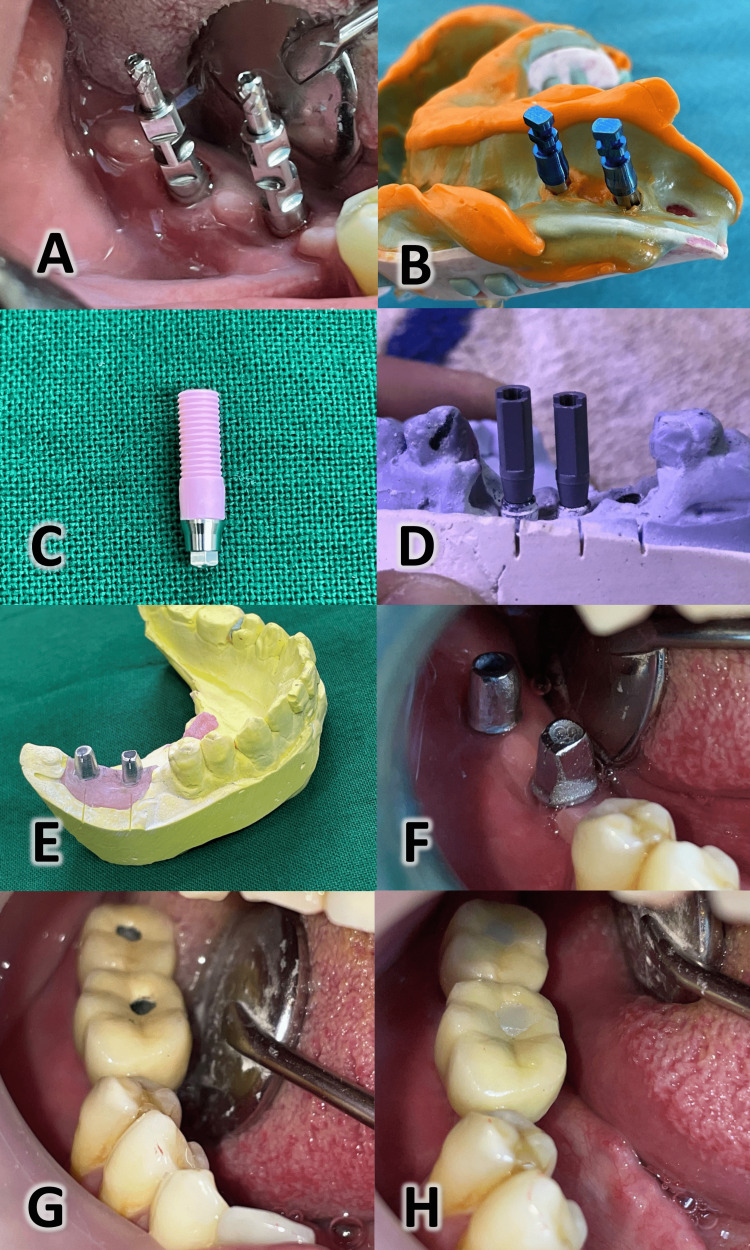
Steps employed in the molded abutment group (A) Transfers used in the open-tray impression technique; (B) open-tray impression; (C) plastic molded abutment; (D) digital scanning used for transferring the implant position to the computer program for the designing of the printed abutment; (E) both abutments after fabrication; (F) trying both abutments in the mouth; (G) fixing the final restoration with screws; (H) closure of the holes with the composite.

The plastic molded abutment, which was available at the implant manufacture, was placed over the analog on the cast to ensure the position of the implant, the abutment, and its relationship with the rest of the teeth. Any necessary modifications to the shape of the plastic abutment to make it suit the location of the implant and its relationship with the adjacent teeth were made, if needed, using wax. The waxed plastic abutment was dismantled, wedged, and poured using a Ni-Cr mixture. Then it was trimmed, making it ready to make the permanent restoration over it. The prostheses were cemented above the abutment using dual-cure resin cement, and then, the final restoration (crown and abutment) was fixed in a screw-retained manner (Figures 5A-5H).

Second group: 3D printed abutments

**Figure 6:**
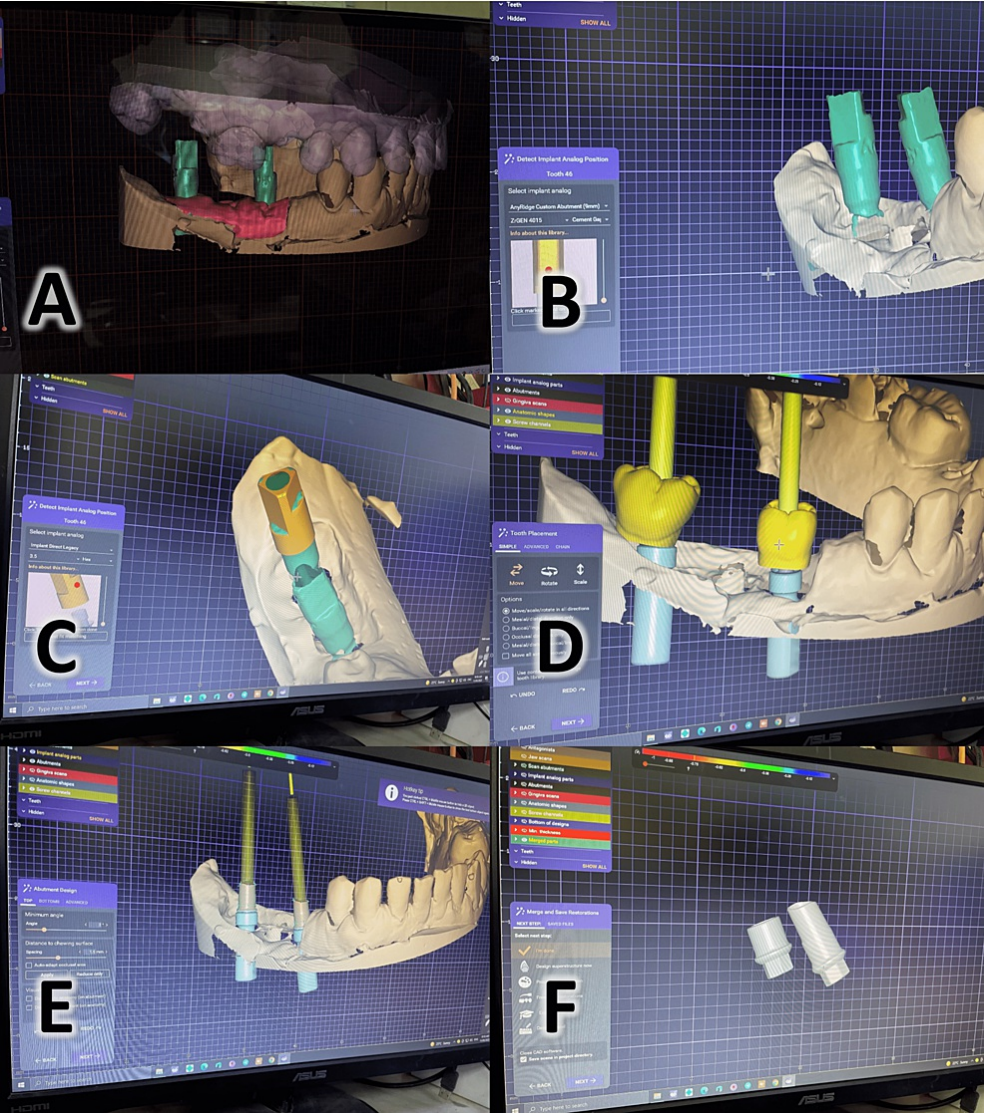
Steps employed in the 3D printed abutment group (A) The marker specific for the implant type after scanning the cast; (B) choosing the implant type from the library of different designs; (C) choosing the digital-analog suitable for the implant type; (D) a virtual design for the final prosthesis above the abutment; (E) designing the custom printed abutment; (F) the final shape of the custom abutment.

A marker or scan body appropriate for the type of implant was placed on the laboratory implant substitute. A digital scan of the marker was done in the lab. The scanned image was transferred to a computer designing software where the prosthesis was designed [18]. Thereafter, the design was virtually sliced and placed with support structures within the build platform in the computer manufacturing software (Figures 6A-6F).

In the printing phase, the final prosthesis was manufactured using a stepwise metal powder supply and a laser fusion process. Finally, heat treatment was performed to relieve the internal stress caused by thermal gradients during the whole 3D printing process, making the abutment ready to make the permanent restoration over it [19]. The prostheses were cemented above the abutment using dual-cure resin cement and then, the final restoration (crown and abutment) was fixed in a screw-retained manner.

Making final prosthesis over both types of abutments

After making the abutments, an experiment was conducted for the abutment inside the mouth to ensure its correct position. The abutments were radiographed using the intra-oral sensor (EzSensor Classic; Vatech, Gyeonggi-do, South Korea). The abutment was placed on the implant analog and photographed using the digital scanner. The metal caps were designed on top of them using exocad DentalCAD software (exocad GmbH, Darmstadt, Germany). Then, they were printed using a 3D laser printer. The porcelain was built on top of the metal caps to give them the shape of the final restoration.

Outcome measures

Radiographic Variables

Preapical radiographs were taken for both groups after fixing the final restoration, using a digital intra-oral sensor (EzSensor Classic), and a special holder in a parallel way, with the same head orientation. The preapical radiographs were used to evaluate the level of the bone around the implant, depending on the marginal bone loss score (MBLS) concept by taking two referring points as described by Koutouzis and Lundgren, using a special computer program (Adobe Photoshop CC 2017; Adobe Inc., San Jose, CA) [16].

**Figure 7:**
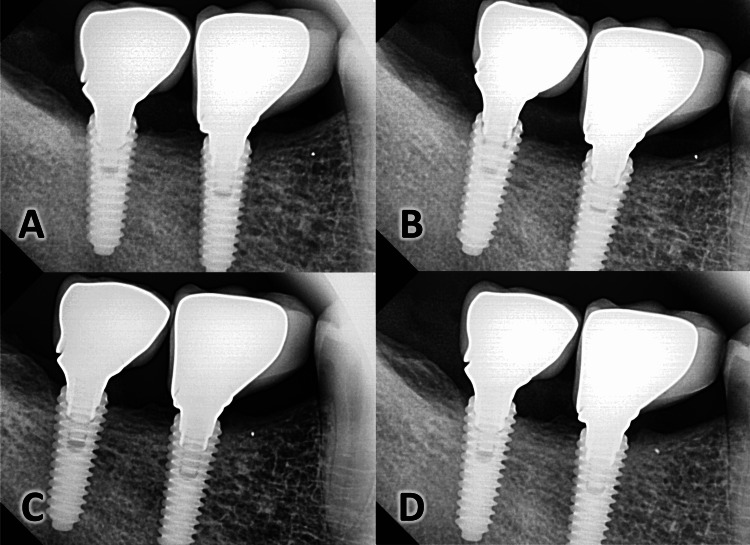
Radiographic assessment of the alveolar bone changes at four assessment times The radiographic appearance of the bone (A) immediately following the fixation of the final prosthesis; (B) after three months; (C) after six months; (D) after one year.

The measurements were taken immediately following fixation and after 3, 6, and 12 months (Figures 7A-7D).

Clinical Variables

The depth of the pocket around the implants was measured from the middle of the mesial and distal surfaces of both types of abutments individually using a special probe (Click-Probe™; Kerr Dental, Orange, CA) immediately following the fixation of the prosthesis and after 3, 6, and 12 months, noting the changes that occurred in the depth of the pocket around the implant in both groups.

Statistical analysis

Data analysis was performed using IBM SPSS Statistics, version 20 (IBM Corp, Armonk, NY), and data were transferred to an Excel spreadsheet (Microsoft). Because of normal distribution, two-sample t-tests were employed to examine intergroup differences. Statistical significance was set at the 0.05 level.

## Results

Baseline sample characteristics

The sample consisted of 32 abutments of 32 implants implanted in 16 patients with the age ranging from 33 to 50 years. The average age of the recruited sample was 45.6 years, with the average age being 44.4 years for males and 46.2 for females (Table 1).

**Table 1:** Baseline sample characteristics (age and sex) N: number of abutments; SD: standard deviation

Variable	Molded abutment (n=16)	Printed abutment (n=16)	Both groups (n=32)
Age (years), mean±SD	44.4±6.9	46.2±2.5	45.6±4.3
Sex, n (%)			
Male	5 (31.3%)	5 (31.3%)	10 (31.3%)
Female	11 (68.8%)	11 (68.8%)	22 (68.8%)

Radiological outcome: change in the bone level around the implants

**Figure 8:**
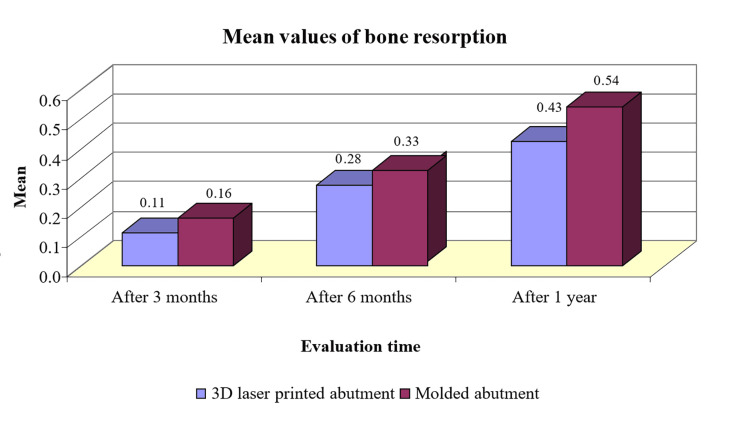
Mean values of bone resorption according to the type of abutment and the assessment time

There were no significant differences in bone resorption mean values between the 3D laser-printed abutment group and the molded abutment group after three- and six-month periods (p>0.05; Table 2). There were significant differences in bone resorption mean values after one year between the 3D laser-printed abutment group and the molded abutment group (p<0.05; Table 2) in the studied sample. Bone resorption mean values in the 3D laser-printed abutment group were lower than those of the molded abutment group after one year (Figure 8).

**Table 2:** Comparisons of the changes that occurred on the level of crystal bone between the two groups according to the assessment time SD: standard deviation An independent-samples t-test was used to detect significant differences in bone resorption (in mm) between the two groups.

Assessment time	Studied group	Mean root resorption	SD	Min.	Max.	Mean difference	t-value	p-value
3 months after cementation	3D laser-printed abutment	0.11	0.10	0	0.4	-0.05	-1.598	0.121
	Molded abutment	0.16	0.08	0	0.3			
6 months after cementation	3D laser-printed abutment	0.28	0.09	0.1	0.5	-0.05	-1.519	0.139
	Molded abutment	0.33	0.10	0.1	0.5			
One year after cementation	3D laser-printed abutment	0.43	0.11	0.2	0.7	-0.12	-3.112	0.004
	Molded abutment	0.54	0.11	0.3	0.7			

Periodontal outcome: change in the probing depth around the implants

**Figure 9:**
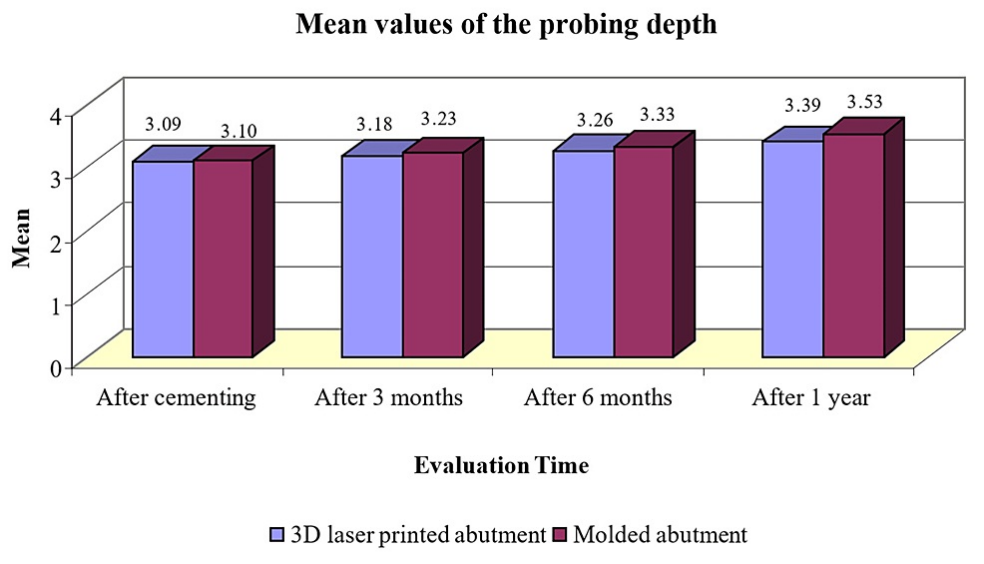
Mean values of the depth of probing according to the type of abutment and the assessment time

After one year of observation, there were significant differences in mean values of the probing depth between the 3D laser-printed abutment group and the molded abutment group (p<0.05; Table 3). The mean values of the probing depth in the 3D laser-printed abutment group were lower than those of the molded abutment group at one year following fixation (Figure 9).

**Table 3:** Comparisons of the changes that occurred in the probing depth between the two groups according to the assessment times SD: standard deviation An independent-samples t-test was used to detect significant differences in the depth of probing between the two groups.

Studied period	Studied group	Mean depth of probing	SD	Min.	Max.	Mean difference	t-value	p-value
Directly after cementing	3D laser-printed abutment	3.09	0.11	3	3.3	-0.01	-0.169	0.867
	Molded abutment	3.10	0.10	3	3.3			
3 months after cementation	3D laser-printed abutment	3.18	0.12	3	3.5	-0.05	-1.291	0.207
	Molded abutment	3.23	0.10	3	3.4			
6 months after cementation	3D laser-printed abutment	3.26	0.13	3.1	3.6	-0.07	-1.701	0.099
	Molded abutment	3.33	0.10	3.2	3.5			
One year after cementation	3D laser-printed abutment	3.39	0.12	3.3	3.7	-0.13	-3.716	0.001
	Molded abutment	3.53	0.08	3.4	3.6			

## Discussion

The amount of bone loss and depth of probing that occurred in both types of abutments after one year can be considered normal and within the normal limits, since the mean bone loss was 0.54 mm and 0.43 mm in the molded abutment and the laser-printed abutment groups, respectively. These mean values are less than 2 mm. At the same time, the probing depth did not exceed the threshold of 4 mm that also showed good periodontal health around the inserted implants [20].

The regression of bone that occurred in the molded abutments was more than that in the printed abutments, despite standardizing the implant measurements and the area of the jaw in which it was implanted. However, the shape and fit of the abutment contributed to an increase in bone resorption. This is because the cast abutments had less diameter in width, and the neckline was simple in contrast to the diameter and shape of the neckline found in the printed abutments. Therefore, the transmission and distribution of forces were better in the printed abutment than in the molded one. The findings of the current study agree with those of another study that indicated that a good adaptation between mechanical components of the implant was essential to minimize biological and prosthetic complications [21].

The risk of bacterial penetration into the implant‐abutment interface has been investigated both in vitro and in vivo. In vitro studies have shown that implant‐abutment connection design characteristics do have an impact. In vivo studies have also confirmed the findings of in vitro studies, and an association of bacterial penetration into the implant‐abutment interface with crestal bone loss has also been shown. We agree with the study of Koutouzis and Lundgren who found that the shape of the junction of the implant and the abutment had an effect on the marginal bone resorption, and it must be designed to prevent the microorganisms from leaking into the contact surface between them and causing bony absorption [16].

In the current study, the depth of probing in the printed abutments was less than that of the molded abutments, and the depth of the probing was related to the level of the bone around the implant. Therefore, any change in the bone would lead to a change in the probing depth; this may be because the better fit of the printed abutments within the implants allowed the transfer of the forces applied to the implant perpendicularly, which allowed maintaining the least degree of bone loss, which is within the international standard natural limits.

The findings of the current work agree with those of a study conducted by Kuo et al. in which the prostheses made using a laser printer achieved better fit and sealing of edges than the prostheses made using the lost-wax technology [22]. Another study found that the abutments made using laser printing were better than the ones made using the lost-wax technique, while some of the molded abutments had a misfit inside the implant that led to the concentration of the occlusal forces in the contact area between the implant and the abutment causing more bone loss [12]. In the traditional casting method, the molten alloy used for crown restoration shrinks upon solidification, and Ni-Cr alloys shrink by as much as 2%. If the molded abutment was not made correspondingly larger than the original wax pattern, the resultant cast will be much smaller. Therefore, for casting abutment, it is necessary to compensate for the solidification-related shrinkage of the alloy used by expanding the mold size to at least equal the shrinkage size. The current results do not go in line with those of Quante et al. who found that the prostheses made by the lost-wax technique had a better fit than the prostheses made from chromium-cobalt using a laser 3D printer [12]. Also, the current results differed from those obtained by Bae et al. who concluded that the 3D laser printing method had more internal incompatibility than the prostheses made using the lost-wax technique [23].

Limitations of the current study

One of the limitations of the current work is the short follow-up period that could have been extended to 18 or 24 months. Another limitation could be the relatively small sample size. Other outcome measures should have been included in the current trial such as the assessment of patient-reported outcomes and possible periodontal complications.

## Conclusions

Within the limitations of the current work, the following conclusions can be made: the amount of bone loss for both types of abutments was within the normal limits. However, the implants that were restored using the 3D printed abutments were better with less bone loss and less probing depth than the implants that were restored using molded abutments.
